# Ameliorating Effect of Pentadecapeptide Derived from *Cyclina sinensis* on Cyclophosphamide-Induced Nephrotoxicity

**DOI:** 10.3390/md18090462

**Published:** 2020-09-09

**Authors:** Xiaoxia Jiang, Zhexin Ren, Biying Zhao, Shuyao Zhou, Xiaoguo Ying, Yunping Tang

**Affiliations:** Zhejiang Provincial Engineering Technology Research Center of Marine Biomedical Products, School of Food and Pharmacy, Zhejiang Ocean University, Zhoushan 316022, China; 15262827607@163.com (X.J.); renzhexin0802@163.com (Z.R.); zhaoby2000@163.com (B.Z.); 15205789697@163.com (S.Z.)

**Keywords:** *Cyclina sinensis*, pentadecapeptide, kidney nephrotoxicity, NF-κB pathway, apoptotic pathway

## Abstract

Cyclophosphamide (CTX) is a widely used anticancer drug with severe nephrotoxicity. The pentadecapeptide (RVAPEEHPVEGRYLV) from *Cyclina sinensis* (SCSP) has been shown to affect immunity and to protect the liver. Hence, the purpose of this study was to investigate the ameliorating effect of SCSP on CTX-induced nephrotoxicity in mice. We injected male ICR mice with CTX (80 mg/kg·day) and measured the nephrotoxicity indices, levels of antioxidant enzymes, malondialdehyde (MDA), inflammatory factors, as well as the major proteins of the NF-κB and apoptotic pathways. Cyclophosphamide induced kidney injury; the levels of kidney-injury indicators and cytokines recovered remarkably in mice after receiving SCSP. The activities of superoxide dismutase (SOD), glutathione peroxidase (GSH-Px), and catalase (CAT) increased, while there was a significant decrease in MDA levels. The kidney tissue damage induced by CTX was also repaired to a certain extent. In addition, SCSP significantly inhibited inflammatory factors and apoptosis by regulating the NF-κB and apoptotic pathways. Our study shows that SCSP has the potential to ameliorate CTX-induced nephrotoxicity and may be used as a therapeutic adjuvant to ameliorate CTX-induced nephrotoxicity.

## 1. Introduction

The kidney is the main excretory organ of the human body and one of the important target organs for studying drug toxicity. It plays an important role in regulating the body’s water, salt, and ion balance [1,2]. Cyclophosphamide (CTX) is widely used as an anticancer drug [3,4], although it can cause side effects such as cardiotoxicity, nephrotoxicity, and hepatotoxicity. The process of CTX-induced renal pathological damage includes apoptosis and necrosis of renal tubular epithelial cells [5], release of inflammatory factors, and mediation of inflammatory response [6,7]. Certain natural products have been shown to mitigate CTX-induced nephrotoxicity during CTX chemotherapy [8,9].

Recently, marine bioactive peptides have attracted the attention of researchers, and many biologically active peptides with specific activities have been discovered from the ocean, all of which exhibited a wide range of biological functions, including liver protection [10]; immune regulation; and antihypertensive, antioxidant, and antibacterial activities [11]. However, reports regarding peptides with kidney-protecting activity are scarce. Previously, we have isolated and purified a pentadecapeptide (RVAPEEHPVEGRYLV) from *Cyclina sinensis* (SCSP) [12]. SCSP increased the activity of lymphocytes and macrophages in the spleen of mice and played a key role in improving cellular immune response and immunity [13]. We also observed that SCSP effectively alleviated CTX-induced liver toxicity in mice and restored the levels of both NF-κB and apoptotic pathway proteins [14]. A preliminary investigation indicated that SCSP showed ameliorating effects against CTX-induced nephrotoxicity. However, until now, the mechanism underlying the ameliorating effect of SCSP on CTX-induced nephrotoxicity in mice is not clear.

Abnormal activation of NF-κB can regulate transcription related to cell proliferation and apoptosis [15,16]. It is well documented that the NF-κB and apoptosis pathways are associated with CTX-induced nephrotoxicity [17]. Kang et al. confirmed that the apoptosis and autophagy signaling pathways in the kidney play an important role in CTX-injured mice [6]. Consequently, in kidney injury, the NF-κB and apoptosis pathways may play an important role and may trigger a severe inflammatory response. Hence, in the present study, we used the NF-κB and apoptotic pathways to elucidate the ameliorating effect of SCSP on CTX-induced renal toxicity in mice. In addition, the nephrotoxicity indices and levels of malondialdehyde (MDA), antioxidant enzymes, and inflammatory factors were also determined. Our results indicate that SCSP is a promising therapeutic adjuvant that can ameliorate CTX-induced nephrotoxicity.

## 2. Results

### 2.1. Effects of SCSP on the Body Weight and Kidney Index of CTX-Induced Mice

In biomedical research, changes in body weight and organ index are important for animal experiments [6]. After the mice were sacrificed, the kidney tissue was excised, and the kidney index was calculated after the tissue was weighed. The findings suggested that the weight of mice in the model group decreased after CTX injection, whereas it increased after SCSP injection. At the same time, the kidney index of the CTX group was significantly higher than that of the control group (*p* < 0.01). However, the kidney index after SCSP treatment was significantly lower than that of the CTX group (*p* < 0.01 and *p* < 0.05), indicating that SCSP could alleviate kidney damage (Table 1).

### 2.2. Effect of SCSP on Nephrotoxicity Marker

**Figure 1:**
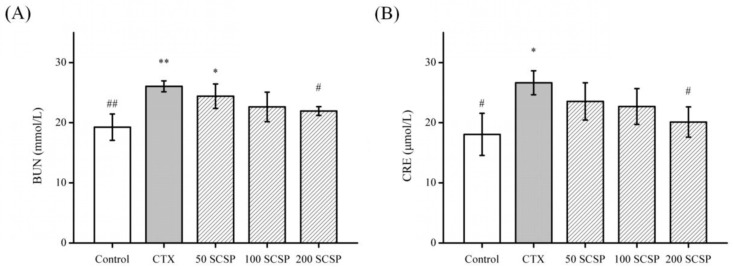
The serum levels of urea (BUN) (**A**) and creatinine (CRE) (**B**): statistical significance was analyzed by ANOVA (Analysis Of Variance). * *p* < 0.05 and ** *p* < 0.01 (vs. control group); # *p* < 0.05 and ## *p* < 0.01 (vs. model group).

Urea (BUN) and creatinine (CRE) are two indicators that are often used to assess kidney injury; hence, determination of BUN and CRE levels can reflect the degree of kidney injury [5,18]. The serum levels of BUN and CRE were determined to assess the ameliorating effect of SCSP against CTX-induced nephrotoxicity (Figure 1). Compared to the control group, the BUN and CRE levels of the model group increased (*p* < 0.05), whereas high-dose SCSP-treated groups (200 mg/kg SCSP) were decreased significantly (*p* < 0.05). Our findings suggested that different doses of SCSP were responsible for kidney recovery to varying extents.

### 2.3. Biochemical Analysis of Liver and Kidney Injury

**Figure 2:**
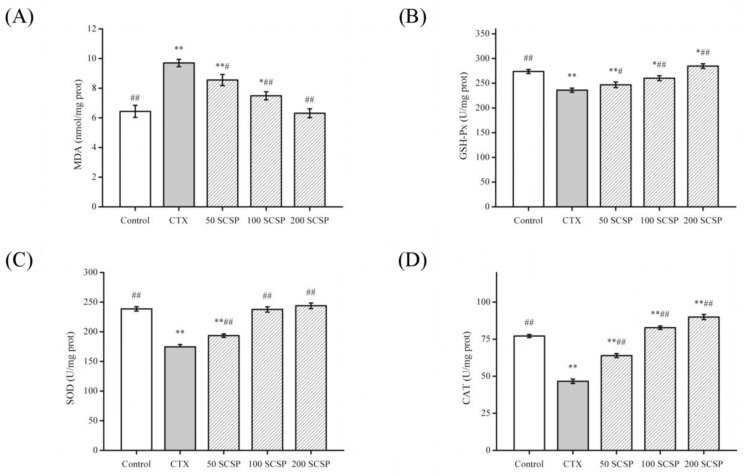
Effect of SCSP (*Cyclina sinensis*) on the levels of renal malondialdehyde (MDA) (**A**), glutathione peroxidase (GSH-Px) (**B**), superoxide dismutase (SOD) (**C**), and catalase (CAT) (**D**) in cyclophosphamide (CTX)-treated mice: statistical significance was analyzed by ANOVA. * *p* < 0.05 and ** *p* < 0.01 (vs. control group); # *p* < 0.05 and ## *p* < 0.01 (vs. model group).

Studies have shown that CTX can change the contents of MDA (lipid peroxidation markers), catalase (CAT), superoxide dismutase (SOD), glutathione peroxidase (GSH-Px) in tissues [19]. To investigate the effect of SCSP on CTX-induced kidney injury, the levels of antioxidant enzymes and MDA were determined. MDA (Figure 2A) levels were significantly higher in the kidneys of CTX-treated mice, while GSH-Px (Figure 2B), SOD (Figure 2C), and CAT (Figure 2D) activities were significantly lower (*p* < 0.01). In addition, after SCSP treatment, GSH-Px, SOD, and CAT contents showed an upward trend (*p* < 0.05). These results showed that SCSP significantly reduced CTX-induced kidney injury to a certain extent and exerted a repair effect on the kidney.

### 2.4. Effect of SCSP on Cytokines

Acute nephrotoxicity is closely related to inflammatory response and induces the expression of various cytokines and chemokines [20], and elevated cytokine levels (such as tumor necrosis factor (TNF)-α, interleukin (IL)-1β, and IL-6) are associated with kidney damage [7,17]. Hence, reducing the level of inflammatory factors and repairing the kidney are important for preventing and treating CTX-induced kidney damage.

**Figure 3:**
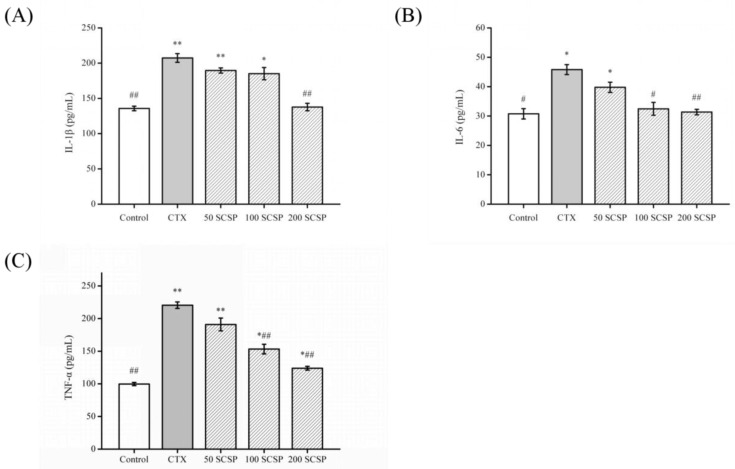
The levels of cytokines in mice kidney tissues: (**A**) interleukin (IL)-1β, (**B**) interleukin (IL)-6, and (**C**) tumor necrosis factor (TNF)-α: statistical significance was analyzed by ANOVA. * *p* < 0.05 and ** *p* < 0.01 (vs. control group); # *p* < 0.05 and ## *p* < 0.01 (vs. model group).

The experimental results are shown in Figure 3. The contents of IL-1β, IL-6, and TNF-α in the kidneys of mice receiving CTX treatment were significantly higher than those in the control group (*p* < 0.05). In contrast, IL-1β, IL-6, and TNF-α levels in the kidney homogenates of mice treated with SCSP (200 mg/kg) decreased markedly compared to the CTX group (*p* < 0.01), which was superior to those in the other treatment groups. Therefore, high doses of SCSP may inhibit secretion of these cytokines and may alleviate CTX-induced renal damage.

### 2.5. Histopathological Analysis

**Figure 4:**
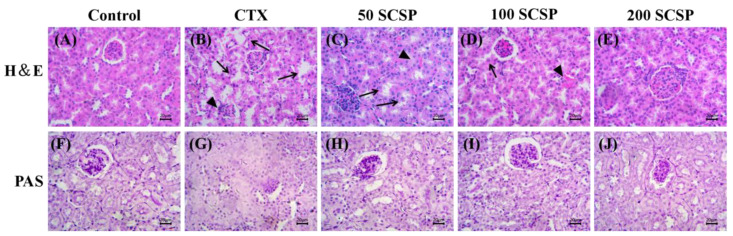
Histopathological analysis for damage in kidney tissues in mice (×400, scale bars of **A**–**J** are 20 μm). Arrows: renal tubular were necrotic and shed, and glomeruli were atrophic; triangle: peritubular and glomerular congestion. CTX represents cyclophosphamide; SCSP represents *Cyclina sinensis*.

Histopathological changes are indicators used to assess renal structural damage [21], and the effect of CTX on kidney histology has been confirmed in previous studies [22]. As shown in the H&E staining results of kidney tissue in Figure 4A–E, compared with control group, renal tubular epithelial cells in the CTX group were necrotic and were shed; the glomeruli were obviously atrophic, desquamated or vacuolated, and peritubular and glomerular congested. Compared to that in the CTX group, SCSP (50 mg/kg) administration reduced CTX-induced renal tissue damage (Figure 4C). Mice treated with 100 mg/kg of SCSP showed reduction in renal tubule degeneration, the renal corpuscles tended to be normal, and the damage was reduced (Figure 4D). In comparison, reduction in kidney damage was obvious in mice treated with 200 mg/kg of SCSP (Figure 4E).

In addition, as shown in the results of the PAS staining of kidney tissue in Figure 4F–J, the glomerular area of the kidney of the CTX group was significantly increased compared with that of the control mice and the mesangial matrix was significantly enlarged. However, as the dose of SCSP increased, the glomerular surface area of the mice in the drug-administered group decreased significantly, and the expansion of the mesangial matrix of the mice in the high-dose treated group also decreased (Figure 4J). Therefore, the results of histopathological indicated that high doses of SCSP provide a certain degree of recovery for the kidney.

### 2.6. Effect of SCSP on CTX-Induced NF-κB Pathway in Mouse Kidney

**Figure 5:**
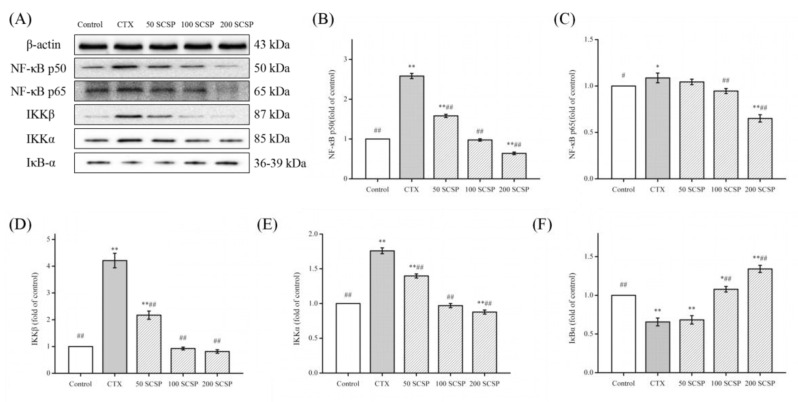
Effect of SCSP (*Cyclina sinensis*) on cyclophosphamide (CTX)-induced kidney inflammation in mice: (**A**) western blot of NF-κB-mediated signaling in the kidney; (**B**) expression of NF-κB p50; (**C**) expression of NF-κB p65; (**D**) expression of IKKβ; (**E**) expression of IKKα; and (**F**) expression of IκBα. Statistical significance was analyzed by ANOVA. * *p* < 0.05 and ** *p* < 0.01 (vs. control group); # *p* < 0.05 and ## *p* < 0.01 (vs. model group).

The NF-κB system is primarily involved in the body’s defense responses, tissue damage, oxidative stress, cell differentiation, and induction of apoptosis [23]. NF-κB mainly refers to the p50/p65 heterodimer, located in the NF-κB/Rel family. Among them, p65 is mainly responsible for the transcriptional activation of genes whereas p50 is responsible for binding to DNA [15]. We have previously demonstrated that the repair role of SCSP on CTX-induced liver in mice occurred via the NF-κB pathway [14]. In the present study, the protein levels of the NF-κB pathway in the CTX-induced kidney increased significantly (Figure 5, *p* < 0.01) whereas CTX-induced mice treated with medium-dose and high-dose SCSP showed significant reduction in the expression of these proteins (*p* < 0.01). However, the IκBα level did not change significantly in the low-dose group. This indicated that SCSP alleviated the CTX-induced kidney injury by inhibiting the NF-κB pathway.

### 2.7. Effect of SCSP on CTX-Induced Kidney Apoptosis

**Figure 6:**
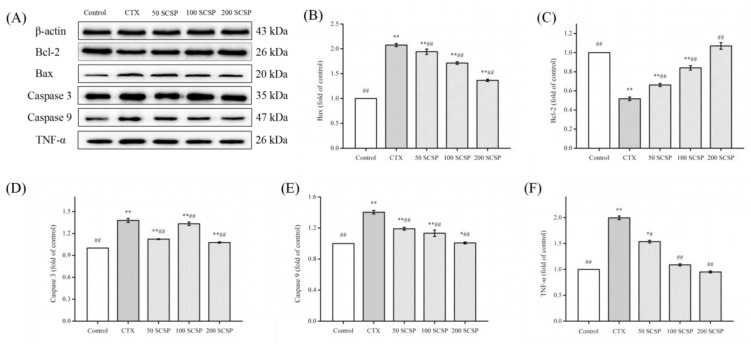
Effect of SCSP (*Cyclina sinensis*) on cyclophosphamide (CTX)-induced kidney apoptosis in mice: (**A**) western blot of signal transduction mediated by the apoptosis pathway in the kidney; (**B**) quantification of Bax expression; (**C**) quantification of Bcl-2 expression; (**D**) quantification of caspase 3 expression; (**E**) quantification of caspase 9 expression; and (**F**) quantification of TNF-α expression. Statistical significance was analyzed by ANOVA. * *p* < 0.05 and ** *p* < 0.01 (vs. control group); # *p* < 0.05 and ## *p* < 0.01 (vs. model group).

Studies have shown that the level of apoptotic protein in mice will change after CTX treatment [6]. To assess the antiapoptotic role of SCSP, the expression of several proteins in the apoptosis pathway were evaluated (Figure 6). Compared to that in the control group, the protein level of Bcl-2 was apparently reduced in the CTX group (Figure 6C, *p* < 0.01). After SCSP treatment, Bcl-2 expression in the kidney was significantly reversed. In contrast, compared to the control group, CTX-induced mice showed significant upregulation of Bax, TNF-α, caspase 3, and caspase 9 protein levels. After SCSP treatment, the protein levels of Bax, TNF-α, caspase 3, and caspase 9 decreased significantly (*p* < 0.01). Our results were consistent with those of previous reports on kidney cell apoptosis and indicated that SCSP can effectively alleviate CTX-induced kidney cell apoptosis.

## 3. Discussion

Cyclophosphamide, a widely used antitumor drug affecting cell morphology and organ function, has been shown to cause nephrotoxicity and kidney tissue damage [24,25]. In this study, we aimed to assess the contribution of SCSP toward regulation of CTX-induced kidney injury. As expected, our results indicated that SCSP can restore the kidney by regulating the NF-κB and apoptotic pathways and significantly restored the levels of BUN, CRE, and cytokines as well as the MDA content and GSH-Px, SOD, and CAT activities, thereby alleviating CTX-induced nephrotoxicity.

Nephrotoxicity and kidney damage are characterized by marked increase in the serum levels of BUN and CRE. To our knowledge, changes in the serum level of BUN can reflect the functional status and excretory function of the kidney. Creatinine is a small molecule that can be filtered through the glomeruli and an increase in serum CRE level indicates reduction in the filtration rate, which could also evaluate renal function [7,17]. In our study, the BUN and CRE levels increased obviously in CTX-treated mice, indicating that CTX is nephrotoxic [5,26]. The increased levels of BUN and CRE after CTX administration may be due to the change in membrane permeability and penetration of the systemic circulation after kidney damage [5]. After SCSP treatment, the BUN and CRE levels gradually returned to normal, indicating that SCSP treatment effectively alleviated CTX-induced nephrotoxicity.

According to the H&E and PAS staining results, CTX caused kidney tissue damage, including necrosis and shedding of kidney tubular epithelial cells, glomerular atrophy, and inflammatory cell invasion in the renal cortex and medulla. After SCSP (200 mg/kg) treatment, renal tubule degeneration decreased and the renal corpuscles tended to be normal, indicating that SCSP was effective in reducing CTX-induced nephrotoxicity.

MDA, the final product of various lipid peroxides produced via lipid peroxidation, is a sensitive indicator of the level of free radical metabolism in the body [27]. In addition, acrolein (a toxic CTX metabolite) can increase the amount of reactive oxygen species (ROS) and can promote lipid peroxidation by combining with glutathione (GSH) [28]. Superoxide dismutase, CAT, and GSH-Px are the most common antioxidants that primarily inhibit or prevent formation of free radicals and ROS in vivo. They are also essential indicators that play important roles in MDA elimination [29]. In our study, SCSP effectively reduced the MDA content and improved antioxidant enzyme activities in the kidney.

Studies have suggested that NF-κB is strongly associated with kidney disease [17,30]. NF-κB is a transcription factor for many inflammatory factors and plays an important role in inflammatory response [31]. NF-κB can control the expression and production of pro-inflammatory cytokines and other inflammatory mediators. Furthermore, NF-κB induces the expression of inflammatory cytokines (TNF-α, IL-1β, and IL-6), amplifies the inflammatory cascade, and is highly activated in some inflammatory disease sites [32,33]. Our results indicated that SCSP regulated the NF-κB signaling pathway in CTX-induced nephrotoxicity. At the same time, SCSP may significantly inhibit secretion of these cytokines, which was consistent with the results of Liu et al. [17].

CTX-induced kidney damage usually involves two forms of apoptosis and cell necrosis [6,34]. Apoptosis is mainly regulated by the Bcl-2 and caspase protein families [35]. TNF-α is also involved in the regulation of a wide spectrum of biological processes including cell proliferation, differentiation, apoptosis, lipid metabolism, and coagulation [36]. Our results showed that SCSP treatment significantly upregulated the expression level of apoptotic protein Bcl-2 (*p* < 0.01) and downregulated Bax, TNF-α, and caspases 3 and 9, thereby reversing the CTX-induced kidney damage. This indicated that SCSP may inhibit the nephrotoxicity caused by CTX via the apoptosis pathway. Overall, our study showed that SCSP can improve CTX-induced nephrotoxicity by inhibiting the NF-κB and apoptotic pathways.

## 4. Materials and Methods

### 4.1. Chemicals and Reagents

SCSP was synthesized by Wuxi MimoTopes Biotechnology (Wuxi, China) [13]. Cyclophosphamide was supplied by Hengrui Medicine (Lianyungang, China). BUN, CRE, GSH-Px, MDA, SOD, CAT determining kits and the H&E staining kit were purchased from Nanjing Jiancheng Bioengineering Institute (Nanjing, China). The ELISA kits and PAS stain kit were purchased from Solarbio Science & Technology Co., Ltd. (Beijing, China). The primary antibodies against β-actin, NF-κB p50, NF-κB p65, IKKα, IKKβ, and IκBα were supplied by Beyotime Biotechnology (Shanghai, China). The monoclonal antibodies against Bcl-2, Bax, caspase 3, and caspase 9 were provided by Cell Signaling Technology Inc. (Beverly, MA, USA). The primary antibodies against TNF-α were supplied by Proteintech Group, Inc. (Wuhan, China). All other reagents were analytically pure.

### 4.2. Animals and Treatment

**Figure 7:**
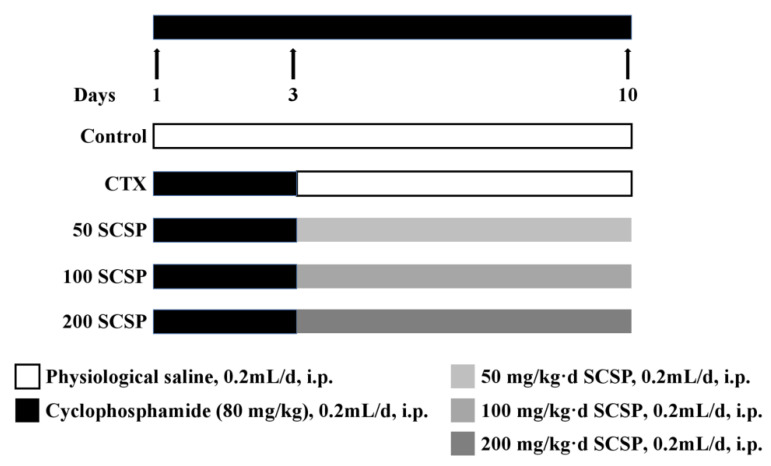
The experimental protocol of mice group and treatment. CTX represents cyclophosphamide; SCSP represents *Cyclina sinensis*.

Male ICR mice (20 ± 2 g, 6 weeks old) were purchased from Zhejiang Academy of Medical Sciences (Hangzhou, China). Experimental procedures were approved by the Animal Ethics Committee of the Committee for Research Ethics and Integrity of Zhejiang Ocean University (Zhoushan, Zhejiang, China, No. SCXK ZHE 2019-0031). All mice were housed in a breeding environment with a standard 12 h daylight/darkness cycle at standard humidity (60 ± 5%) and room temperature (23 ± 2 °C). We fed a commercial pellet diet with free access to sterilized water under a pathogen-free environment. After being adapted to feeding for one week, 50 mice were randomly divided into 5 groups: control, model (CTX, 80 mg/kg), 50 SCSP (SCSP, 50 mg/kg), 100 SCSP (SCSP, 100 mg/kg), and 200 SCSP (SCSP, 200 mg/kg) (Figure 7). Except for the control group, all groups were injected with CTX (80 mg/kg b.wt, dissolved in saline, 0.2 mL) for 3 consecutive days. From the fourth day, SCSP was injected intraperitoneally into each drug group (50, 100, and 200 mg/kg b.wt, 0.2 mL) for 7 consecutive days and an equal volume of saline was injected into the model group. Mice in the control group were injected with an equal volume of normal saline once a day for 10 days. Upon completion of the treatment period, the mice were killed by neck dislocation, and intact kidneys were removed and weighed, some of which were used for subsequent biochemical and pathology experiments. The kidney index was calculated as follows: kidney index (mg/g) = kidney weight/body weight.

### 4.3. Sample Collection and Preparation

The kidneys were dissected and weighed, and the kidney weight ratio was represented as a percentage of kidney weight to body weight. Partial kidney samples were fixed in 10% neutral formalin buffer for 24 h for histopathological analysis. Other kidney samples were partly ground into homogeneous slurry (*w*/*v*, 1:9), and the supernatant was used for biochemical analysis; a part of the supernatant was stored at −80 °C for western blotting. The protein content was determined per the instructions of the BCA kit (Solarbio, Beijing, China).

### 4.4. Measurement of Kidney-Related Parameters

At the end of the study, the serum was separated from the retroorbital whole blood. The serum renal function markers, including BUN and CRE, were measured according to Nanjing Jiancheng’s (Nanjing, China) instructions. The renal tissue was immediately dissected and weighed. The tissue homogenate (10% renal tissue within normal saline, *g*/*g*) was prepared from frozen renal tissues. After centrifuging, the supernatant of the tissue homogenate was collected to measure the MDA level and the GSH-Px, SOD, CAT activities according to the manufacturer’s instructions. The cytokine levels in kidney homogenates were determined per the instructions of Solarbio (Beijing, China).

### 4.5. Histopathological Analysis

The immobilized kidney tissues were embedded with paraffin, and 5-μm-thick sections were excised from the paraffin blocks. H&E staining and PAS staining were performed according to a standard protocol and were observed under a light microscope (Olympus CX31, Tokyo, Japan).

### 4.6. Western Blot Analysis

Kidney tissues were ground with liquid nitrogen and treated with a protease inhibitor to extract the total protein in RIPA buffer. The BCA analysis kit was used to determine protein concentrations. Thirty micrograms of protein were separated using 12% SDS-PAGE, and protein blotting was performed as described previously [14]. Finally, the target bands were detected using an enhanced chemiluminescence kit (ECL) and analyzed using the Alphaview SA software for Fluor Chem FC3 (ProteinSimple, San Jose, CA, USA). β-actin antibody was used as the control.

### 4.7. Statistical Analysis

The SPSS software (version 22.0) was used to analyze the data, and all experimental data were presented as mean ± standard deviation. Statistical significance was analyzed using one-way analysis of variance (ANOVA), followed by the least significant difference (LSD) test as the post hoc test. Statistically, difference at *p* < 0.05 was considered significant while difference at *p* < 0.01 was considered particularly significant. 

## 5. Conclusions

Our results demonstrated that SCSP exerted a potential ameliorating effect against CTX-induced nephrotoxicity, which was reflected in inhibition of the activities of antioxidant enzymes and markers in the kidney and in reduction of inflammation. Furthermore, SCSP also repaired the kidney by restoring the protein levels of the members of the NF-κB and apoptotic signaling pathways. Taken together, our results indicated that SCSP has the potential to ameliorate CTX-induced nephrotoxicity and may be used as a therapeutic adjuvant to ameliorate CTX-induced nephrotoxicity.

## Figures and Tables

**Table 1:** Effect of *Cyclina sinensis* (SCSP) on cyclophosphamide (CTX)-induced mouse kidney index.

Group	Initial Weight (g)	Final Weight (g)	Body Weight Gain (g)	Kidney Weight (g)	Kidney Index (mg/g)
Control	20.47 ± 1.52	23.84 ± 1.74	2.49 ± 0.49	0.30 ± 0.04	1.44 ± 0.05 ^##^
Model	21.04 ± 1.16	22.68 ± 2.57	2.59 ± 1.59	0.34 ± 0.03	1.65 ± 0.02 **
50 SCSP	21.17 ± 1.04 ^##^	23.19 ± 1.04 **	3.10 ± 0.66 **	0.33 ± 0.04	1.59 ± 0.02 *
100 SCSP	21.78 ± 1.35 ^##^	23.38 ± 1.34 **^#^	2.70 ± 0.26 ^#^	0.32 ± 0.04	1.53 ± 0.03 ^##^
200 SCSP	21.10 ± 1.49 ^##^	23.34 ± 1.87 **^#^	2.97 ± 0.70 ^#^	0.31 ± 0.04	1.51 ± 0.04 ^##^

Data were shown as mean ± SD (*n* = 10). Statistical significance was analyzed by ANOVA (Analysis Of Variance). * *p* < 0.05 and ** *p* < 0.01 (vs. control group); ^#^ *p* < 0.05 and ^##^ *p* < 0.01 (vs. model group).
